# Food Consumption and Risk of Islet Autoimmunity and Type 1 Diabetes in Children at Increased Genetic Susceptibility for Type 1 Diabetes

**DOI:** 10.1016/j.tjnut.2024.09.018

**Published:** 2024-09-21

**Authors:** Suvi M Virtanen, Essi J Peltonen, Leena Hakola, Sari Niinistö, Hanna-Mari Takkinen, Suvi Ahonen, Mari Åkerlund, Ulla Uusitalo, Markus Mattila, Tuuli EI Salo, Jorma Ilonen, Jorma Toppari, Riitta Veijola, Mikael Knip, Jaakko Nevalainen

**Affiliations:** 1 Unit of Health Sciences, Faculty of Social Sciences, Tampere University, Tampere, Finland; 2 Wellbeing Services County of Pirkanmaa, Tampere University Hospital, Tampere, Finland; 3 Department of Public Health and Welfare, Finnish Institute for Health and Welfare, Helsinki, Finland; 4 Health Informatics Institute, Morsani College of Medicine, University of South Florida, Tampa, FL, United States; 5 Institute of Biomedicine, Immunogenetics Laboratory, University of Turku, Turku, Finland; 6 Institute of Biomedicine, Research Centre for Integrative Physiology and Pharmacology, and Centre for Population Health Research, University of Turku, Turku, Finland; 7 Department of Pediatrics, Turku University Hospital, Turku, Finland; 8 Department of Paediatrics, Research Unit of Clinical Medicine, Medical Research Centre, University of Oulu, Oulu, Finland; 9 Department of Children and Adolescents, Oulu University Hospital, Oulu, Finland; 10 Research Program for Clinical and Molecular Metabolism, Faculty of Medicine, University of Helsinki, Helsinki, Finland; 11 Department of Pediatrics, Tampere University Hospital, Tampere, Finland

**Keywords:** child, islet autoimmunity, diabetes mellitus, type 1, diet, food consumption, multivariate joint models, survival analysis

## Abstract

**Background:**

Prospective longitudinal evidence considering the entire childhood food consumption in relation to the development of islet autoimmunity (IA or) type 1 diabetes is lacking.

**Objectives:**

We studied the associations of consumption of various foods and their combinations with IA and type 1 diabetes risk.

**Methods:**

Children with genetic susceptibility to type 1 diabetes born in 1996–2004 were followed from birth up to ≤6 y of age in the prospective birth cohort type 1 diabetes prediction and prevention study (*n* = 5674). Exposure variables included 34 food groups covering the entire diet based on repeated 3-d food records at ages 3 mo to 6 y. Endpoints were islet cell antibodies plus biochemical IA (*n* = 247), multiple biochemical IA (*n* = 206), and type 1 diabetes (*n* = 94). We analyzed associations between longitudinally observed foods and risk of IA/type 1 diabetes using a Bayesian approach to joint models in 1-food and multi-food models adjusted for energy intake, sex, human leukocyte antigen genotype, and familial diabetes.

**Results:**

The final multi-food model for islet cell antibodies plus biochemical IA included oats [hazard ratio (HR): 1.09; 95% credible interval (CI): 1.04, 1.14], banana (HR: 1.07; 95% CI: 1.03, 1.11), and cruciferous vegetables (HR: 0.83; 95% CI: 0.73, 0.94). The final model for multiple biochemical IA included, in addition to the above-mentioned foods, fermented dairy (HR: 1.42; 95% CI: 1.12, 1.78) and wheat (HR: 1.10; 95% CI: 1.03, 1.18). The final multi-food model for type 1 diabetes included rye (HR: 1.27; 95% CI: 1.07, 1.50), oats (HR: 1.15; 95% CI: 1.03, 1.26), fruits (HR: 1.05; 95% CI: 1.01, 1.09), and berries (HR: 0.67; 95% CI: 0.50, 0.93).

**Conclusions:**

Higher consumption of oats, gluten-containing cereals, and fruits was associated with increased that of cruciferous vegetables with decreased risk of several type 1 diabetes-related endpoints when considering all the foods in combination. Further etiological and mechanistic studies are warranted.

## Introduction

The increasing incidence of type 1 diabetes causes a large human and economic burden [1]. In genetically susceptible individuals, environmental factors such as virus infections, diet, environmental chemicals, microbiota, and their interactions with each other and genes are thought to affect the development of islet autoimmunity (IA) and type 1 diabetes [2].

Lampousi et al. [3] concluded, based on their systematic review and meta-analysis of retrospective and prospective human evidence, that breastfeeding and late introduction of gluten, fruit, and cow milk may reduce risk of type 1 diabetes, whereas high intake of cow milk in childhood may increase risk. According to the type 1 diabetes prediction and prevention (DIPP) study on the foods consumed in childhood, fish, cruciferous vegetables, and berries are associated inversely, whereas dairy products, meat, gluten-containing cereals, oats, and fruits are associated directly with risk of IA, type 1 diabetes, and/or progression from IA to type 1 diabetes [[4], [5], [6], [7]]. Most of these foods have not yet been analyzed and reported by other prospective studies. The findings regarding dairy products are supported by several cohorts [3,8], and childhood gluten intake was associated with type 1 diabetes risk also in another cohort [9].

Prompted by our previous findings in the DIPP cohort using the joint model for longitudinal and time-to-event data for single foods [[4], [5], [6], [7]], we set out to assess prospectively whether the observed associations of single foods with type 1 diabetes-related endpoints are additive or confounded by each other. By using newly introduced multivariate variants of the joint model, we explored which foods and which food combinations are related to the disease process leading to type 1 diabetes.

## Methods

### Study design and participants

Children born between September 1996 and September 2004 in Oulu and Tampere University Hospitals in Finland were screened from cord blood for genes indicating high or moderate human leukocyte antigen (HLA) -conferred risk of type 1 diabetes as a part of the DIPP prospective birth cohort (see Supplemental Methods and Supplemental Figure 1) [10]. Children were screened for islet cell antibodies (ICA) at intervals of 3–12 mo and selectively for biochemical autoantibodies as described before (Supplemental Methods) [10]. Altogether, 5626 children had food record and IA data, 5674 children had food record and type 1 diagnosis data, and 505 children had food record data at or after repeated positivity to ≥1 autoantibody and were included in the analyses (Supplemental Figure 1).

### IA and type 1 diabetes

The main endpoints were 2 IA endpoints and clinical type 1 diabetes. A secondary endpoint was progression from IA to type 1 diabetes. Two IA endpoints were used: *1*) repeated positivity for ICA and ≥1 biochemical autoantibody (insulin autoantibodies, glutamic acid decarboxylase autoantibodies, or islet antigen-2 autoantibodies) or having type 1 diabetes (ICA plus biochemical IA endpoint); and *2*) repeated positivity for ≥2 biochemical autoantibodies or having type 1 diabetes (multiple biochemical IA endpoint). The first IA endpoint is based on the DIPP follow-up protocol, whereas the second one is more comparable to other studies [11]. Event times for children with IA were set to the middle of the time interval between the last IA-negative and the IA-positive measurement to approximate the interval censoring in the IA endpoints. Type 1 diabetes information was obtained from Oulu and Tampere University Hospitals and the Finnish pediatric diabetes registry. In progression analysis, risk of type 1 diabetes was assessed among children who were repeatedly positive for ≥1 autoantibody.

### Dietary methods

The main exposure variables included 34 ingredient-based food groups, which covered the entire diet (Supplemental Table 1). The food consumption information was collected using a 3-d food record at the age of 3 and 6 mo, and 1, 2, 3, 4, 5, and 6 y. The research nutritionists trained and motivated the research personnel [12] (Supplemental Methods). The annually updated national food composition database Fineli and in-house dietary calculation software Finessi, connected to it, summarized the intake of various food ingredients, for example, milk from different dairy products and foods containing milk. A food composition database developed for commercial infant foods and infant formulas enabled detailed coding of these foods.

### Covariates

Information on the child’s sex, diabetes status of the first-degree relatives, and maternal vocational education (none, vocational, secondary vocational, university studies, or degree) was collected by a structured questionnaire after the delivery, and information on the duration of breastfeeding at the study visits. Information on the age at introduction of new foods was asked with structured questionnaires during infancy. Age at introduction of solid foods was used in the current study (in thirds). All these variables were used as baseline variables in the analysis.

### Statistical methods

Univariate and multivariate joint models that combine longitudinal and time-to-event data into a single model [13] were used to investigate the association between consumption of different foods and risk of IA and type 1 diabetes. All the 34 food groups were analyzed (Supplemental Table 1**)**. Out of the 15 main food groups, 6 groups had subgroups. Longitudinal intake of foods from 3 mo until 6 y was modeled using piecewise natural cubic splines with 3 knots in the linear mixed effects submodels. The time-to-event submodel was built using the structure of the Cox proportional hazards regression model. The children were followed until the appearance of type 1 diabetes-related endpoint, last endpoint measurement, or the age of 6 y. The longitudinal and time-to-event submodels were fitted simultaneously and linked together by using a current value association structure. The model estimated a complete food consumption trajectory over the entire study period for each child, even if a series of repeated dietary measurements were incomplete. Thus, children with some missing records could still be included in the analyses, which enabled higher statistical power and avoidance of selection bias. Joint models were estimated within a Bayesian framework using Markov chain Monte Carlo algorithms. Statistical methods, including secondary analyses with progression endpoint, are described in more detail in Supplemental Methods.

The analyses were implemented by the following steps separately for each endpoint. Step 1: 1-food joint models were analyzed for each energy-adjusted food item for all type 1 diabetes-related endpoints. Step 2: All foods out of the 34 foods associated with the endpoint (criteria *P* < 0.1) at Step 1 were selected for the preliminary multi-food model, and the corresponding model was fitted. Step 3: All foods that were associated with the endpoint (criteria *P* < 0.05) in Step 2 preliminary multi-food model were included in the final multi-food model. In addition, those foods that dropped from Step 2 to Step 3 were included 1 by 1 in the model to re-verify their significance (*P* < 0.05). Step 4: The final multi-food model incorporating the foods selected on the Step 3 model was fitted. When both the main food group and its subgroup/s showed associations with the disease endpoints at Step 1, we considered whether the association was driven by the specific subgroup or by several subgroups. If only 1 subgroup showed an association (*P* < 0.1), it was chosen for the preliminary multi-food model. If 2 or more subgroups showed a parallel association (*P* < 0.1), the main group was chosen for the preliminary multi-food model.

All the models were adjusted for the child’s sex (boy/girl), human leukocyte antigen DQ beta 1 isotype (HLA-DQB1)-associated disease risk (moderate/high), and familial diabetes (no/yes), which were used as baseline variables. In addition, the models were adjusted for energy intake. The IA endpoints were also analyzed without energy adjustment. Energy adjustment was implemented using a multivariate nutrient density method [14]: absolute food consumption was divided by the total energy intake [megajoules (MJ)], and the energy-adjusted consumption as well as the total energy intake were included in a model as longitudinal covariates. The progression endpoint was additionally adjusted for age at seroconversion to the first autoantibody, which was used as a baseline variable in the analysis.

We report the posterior mean estimates as hazard ratios (HRs) with 95% credible intervals (CIs). The HR is provided for a 1-unit (100 g, 10 g, 1 g, 20 g/MJ, 1 g/MJ, 250 mg/MJ) increase in the current value of the food consumption. To control for type 1 errors due to multiple testing, only *P* < 0.01 was interpreted as convincing evidence of the association in the final multi-food models. Analyses were performed using the jm function from the JMbayes2 package [15] in R version 4.3.0 [16].

As a sensitivity analysis, the final multi-food models were adjusted for energy-adjusted intake of fat, n–3 fatty acids, protein, and/or dietary fiber (selected depending on their associations with the endpoints) as well as breastfeeding at the age of 6 mo (no/yes) as these dietary factors have shown associations with IA and/or type 1 diabetes [3,4,17,18]. Also, to study whether a child’s sex, HLA-DQB1-associated disease risk, or breastfeeding at 6 mo of age modified the association between food consumption and type 1 diabetes-related endpoints, the interaction term between those factors, respectively, and food consumption was added to the 1-food model. To study whether the association between the food consumption and the endpoints of the foods included in the final multi-food models was time-varying, the interaction term between the consumption and a natural cubic spline of age with 1 knot at 3 y was added in the 1-food model, and the model was compared to the original 1 using Deviance and Watanabe-Akaike information criteria, and log pseudo-marginal likelihood. If ≥2 of the criteria suggested the existence of a time-varying coefficient, the association was visually inspected. For the type 1 diabetes endpoints, we performed sensitivity analyses by excluding food consumption data 18 mo before diagnosis, during the time when changes in glucose metabolism prior to clinical disease have been observed [19,20]. These analyses were implemented for foods included in the final multi-food models. In addition to the final multi-food models, we fitted for each endpoint a fixed multi-food model, including foods that were shown to be the most important for all the endpoints (energy-adjusted oats, gluten-containing cereals, fruits, and cruciferous vegetables).

### Ethical aspects

The study adheres to the declaration of Helsinki, and the local ethics committees approved the study protocol. Families gave their written informed consent for the genetic testing of the newborn infant and for their participation in the follow-up study.

## Results

The distribution of the participating children according to main baseline covariates and breastfeeding, and risk of ICA plus biochemical IA, multiple biochemical IA, and type 1 diabetes associated with them are presented in Table 1. Being breastfed at 6 mo of age was associated with a decreased risk of both the IA endpoints (*P* < 0.001). The 34 food groups used in the analysis are described in Supplemental Table 1. For each food group, we present the proportion of users and mean consumption by age: 3–12 mo and 2–6 y (Supplemental Table 1).

**TABLE 1:** Distribution of participating children according to baseline characteristics and duration of breastfeeding and their associations with the risk islet autoimmunity and type 1 diabetes by age 6 y.

Characteristic	Islet autoimmunity cohort (*n* = 5626)					Type 1 diabetes cohort (*n* = 5674)		
	All, *n* (%)	ICA plusbiochemicalIA^1^, *n* (%)	HR(95% CI)	MultiplebiochemicalIA^1^, *n* (%)	HR(95% CI)	All,*n* (%)	Type 1diabetes^2^,*n* (%)	HR(95% CI)
Child sex^3^								
Boys	2988 (53.1)	148 (5.0)	1	126 (4.2)	1	3010 (53.0)	54 (1.8)	1
Girls	2638 (46.9)	99 (3.8)	0.74 (0.57, 0.95)	80 (3.0)	0.70 (0.53, 0.92)	2664 (47.0)	40 (1.5)	0.82 (0.55, 1.24)
HLA-DQB1-conferred risk^3^								
Moderate	4524 (80.4)	170 (3.8)	1	137 (3.0)	1	4565 (80.5)	59 (1.3)	1
High	1102 (19.6)	77 (7.0)	1.87 (1.43, 2.45)	69 (6.3)	2.07 (1.55, 2.77)	1109 (19.5)	35 (3.2)	2.43 (1.60, 3.69)
Familial diabetes^3^								
No	5080 (90.3)	211 (4.2)	1	171 (3.4)	1	5125 (90.3)	78 (1.5)	1
Yes	333 (5.9)	30 (9.0)	2.10 (1.43, 3.09)	29 (8.7)	2.51 (1.69, 3.72)	333 (5.9)	14 (4.2)	2.74 (1.55, 4.84)
Missing information	213 (3.8)	6 (2.8)	0.71 (0.32, 1.61)	6 (2.8)	0.88 (0.39, 1.99)	216 (3.8)	2 (0.9)	0.60 (0.15, 2.45)
Breastfed at 6 mo^4^								
No	2130 (37.9)	106 (5.0)	1	89 (4.2)	1	2141 (37.7)	42 (2.0)	1
Yes	3254 (57.8)	132 (4.1)	0.76 (0.59, 0.98)	107 (3.3)	0.74 (0.56, 0.98)	3262 (57.5)	45 (1.4)	0.73 (0.48, 1.12)
Missing information	242 (4.3)	9 (3.7)	2.44 (1.24, 4.83)	10 (4.1)	3.22 (1.67, 6.21)	271 (4.8)	7 (2.6)	1.40 (0.63, 3.13)

Abbreviations: CI, credible interval; HLA, human leukocyte antigen; HR, hazard ratio; IA, islet autoimmunity; ICA, islet cell antibodies; DQB1, DQ beta 1 isotype.

1 During the 6-y follow-up, 247 children (4.4%) developed the ICA plus biochemical IA endpoint at a median [first and third quartile (Q_1_, Q_3_)] age of 2.5 (1.3, 3.6) y and 206 (3.7%) multiple biochemical IA at a median (Q_1_, Q_3_) age of 2.5 (1.3, 3.6) y.

2 Of the 5674 children with food records and type 1 diabetes data available, 94 children (1.7%) developed type 1 diabetes during the 6-y follow-up at a median (Q_1_, Q_3_) age of 4.0 (2.9, 5.0) y.

3 Estimates are from the Cox proportional hazards model, including child sex, HLA-DQB1-conferred risk, and familial diabetes as baseline covariates.

4 Estimates are from the Cox proportional hazards model, including child sex, HLA-DQB1-conferred risk, familial diabetes, and breastfeeding at 6 mo as baseline covariates.

As the risks of both the IA endpoints were associated in a fairly similar way with absolute (Supplemental Table 2) and energy-adjusted food consumption (TABLE 2, TABLE 3), we used only energy-adjusted food consumption in all the further analyses. For type 1 diabetes endpoints, we used energy-adjusted food consumption as the energy intake may increase near the disease diagnosis due to the disturbed metabolism. The use of energy adjustment for all the endpoints is justified also by the fact that energy varies substantially by age and sex in young children.

**TABLE 2:** Risk of islet cell antibodies plus biochemical islet autoimmunity by age 6 y (*n* = 247) associated with food consumption (*n* = 5626)^1^.

Energy and foods^2^: main groupsand subgroups	One-food models^2^		Preliminary multi-food model^3^		Final multi-food model^4^	
	HR (95% CI)	*P* value	HR (95% CI)	*P* value	HR (95% CI)	*P* value
Energy intake (MJ)	1.04 (0.99, 1.09)	0.115	0.87 (0.70, 1.07)	0.193	0.97 (0.80, 1.16)	0.719
Dairy products						
Fermented dairy (20 g/MJ)	1.24 (0.99, 1.54)	0.059	1.13 (0.87, 1.43)	0.337		
Meat and meat products (1 g/MJ)	1.04 (1.00, 1.07)	0.037				
Meat products (1 g/MJ)	1.06 (0.99, 1.12)	0.076	1.05 (0.98, 1.12)	0.151		
Gluten-containing cereals (1 g/MJ)	1.06 (1.01, 1.11)	0.007	1.04 (0.99, 1.09)	0.105		
Wheat (1 g/MJ)	1.06 (1.00, 1.12)	0.047				
Rye (1 g/MJ)	1.08 (0.99, 1.18)	0.091				
Oats (1 g/MJ)	1.10 (1.04, 1.15)	<0.001	1.09 (1.03, 1.14)	0.001	1.09 (1.04, 1.14)	<0.001
Fruits (1 g/MJ)	1.02 (1.00, 1.04)	0.020				
Banana (1 g/MJ)	1.08 (1.04, 1.12)	<0.001	1.06 (1.02, 1.11)	0.009	1.07 (1.03, 1.11)	0.001
Vegetables						
Cruciferous vegetables (1 g/MJ)	0.83 (0.72, 0.95)	0.007	0.84 (0.73, 0.96)	0.009	0.83 (0.73, 0.94)	0.003
Onions (1 g/MJ)	1.20 (0.98, 1.44)	0.077	1.00 (0.79, 1.25)	0.994		
Potato (1 g/MJ)	1.02 (1.00, 1.04)	0.051	1.01 (0.99, 1.04)	0.316		

Abbreviations: CI, credible interval; HR, hazard ratio; MJ, megajoule.

1 HR and 95% CI are from the joint model with longitudinally assessed food consumption exposure (3 mo to 6 y). All the 1-food and multi-food models were adjusted for sex, genotype, and familial diabetes at baseline. In addition, the energy adjustment was done in all the models by dividing foods by energy and including energy in the model.

2 All the foods out of the 34 foods that were associated with the endpoint (*P* < 0.1) in 1-food models were selected for this table. Main food group names are presented for all the subgroups that remained in the analyses.

3 Foods significantly associated with the endpoint (*P* < 0.1) were entered into the preliminary multi-food model. If both the main food group and its subgroup were associated with the endpoint, subgroup was used (e.g., meat products), but if several subgroups were associated with the endpoint, main food group was used (e.g., gluten-containing cereals).

4 The final multi-food model included all the foods that were significant (*P* < 0.05) in the preliminary multi-food model and when included in the final model. Those variables that dropped from the analysis were further individually added to the model, but none of them was significantly (*P* < 0.05) associated with the endpoint (data not shown).

**TABLE 3:** Risk of multiple biochemical islet autoimmunity by age 6 y (*n* = 206) associated with food consumption (*n* = 5626)^1^.

Energy and foods^2^: main groupsand subgroups	One-food models^2^		Preliminary multi-food model^3^		Final multi-food model^4^	
	HR (95% CI)	*P* value	HR (95% CI)	*P* value	HR (95% CI)	*P* value
Energy intake (MJ)			0.87 (0.68, 1.11)	0.256	0.95 (0.76, 1.17)	0.622
Dairy products (20 g/MJ)	1.06 (1.01, 1.12)	0.028				
Fermented dairy (20 g/MJ)	1.52 (1.21, 1.86)	<0.001	1.39 (1.08, 1.74)	0.014	1.42 (1.12, 1.78)	0.006
Meat and meat products (1 g/MJ)	1.06 (1.02, 1.10)	0.001				
Red meat (1 g/MJ)	1.06 (1.01, 1.11)	0.014	0.99 (0.93, 1.05)	0.764		
Gluten-containing cereals (1 g/MJ)	1.08 (1.03, 1.13)	<0.001				
Wheat (1 g/MJ)	1.12 (1.05, 1.19)	0.002	1.10 (1.02, 1.18)	0.013	1.10 (1.03, 1.18)	0.006
Oats (1 g/MJ)	1.12 (1.06, 1.17)	<0.001	1.11 (1.05, 1.17)	<0.001	1.12 (1.06, 1.18)	<0.001
Fruits (1g/MJ)	1.03 (1.00, 1.05)	0.026				
Banana (1 g/MJ)	1.09 (1.04, 1.13)	0.001	1.06 (1.00, 1.11)	0.032	1.07 (1.02, 1.12)	0.007
Juice (1 g/MJ)	1.01 (1.00, 1.02)	0.071	1.01 (1.00, 1.02)	0.114		
Vegetables						
Fruit vegetables (1 g/MJ)	1.04 (1.01, 1.08)	0.026	1.03 (0.98, 1.06)	0.226		
Cruciferous vegetables (1 g/MJ)	0.80 (0.69, 0.94)	0.003	0.82 (0.69, 0.96)	0.010	0.82 (0.70, 0.95)	0.007
Onions (1 g/MJ)	1.40 (1.15, 1.68)	<0.001	1.12 (0.84, 1.44)	0.417		
Potato (1 g/MJ)	1.02 (1.00, 1.05)	0.048	1.02 (0.99, 1.05)	0.196		

Abbreviations: CI, credible interval; HR, hazard ratio; MJ, megajoule.

1 HR and 95% credible interval are from the joint model with longitudinally assessed food consumption exposure (3 mo to 6 y). All the 1-food and multi-food models were adjusted for sex, genotype, and familial diabetes at baseline. In addition, the energy adjustment was done in all the models by dividing foods by energy and including energy in the model.

2 All the foods out of the 34 foods that were associated with the endpoint (*P* < 0.1) in 1-food models were selected for this table. Main food group names are presented for all the subgroups that remained in the analyses.

3 Foods significantly associated with the endpoint (P < 0.1) were entered into the preliminary multi-food model. If both the main food group and its subgroup were associated with the endpoint, the subgroup was used (e.g., fermented dairy), but if several subgroups were associated with the endpoint, the main food group was used (did not occur in this table).

4 The final multi-food model included all the foods that were significant (*P* < 0.05) in the preliminary multi-food model and when included in the final model. Those variables that dropped from the analysis were further individually added to the model, but none of them was significantly (*P* < 0.05) associated with the endpoint (data not shown).

### ICA plus biochemical IA

**FIGURE 1:**
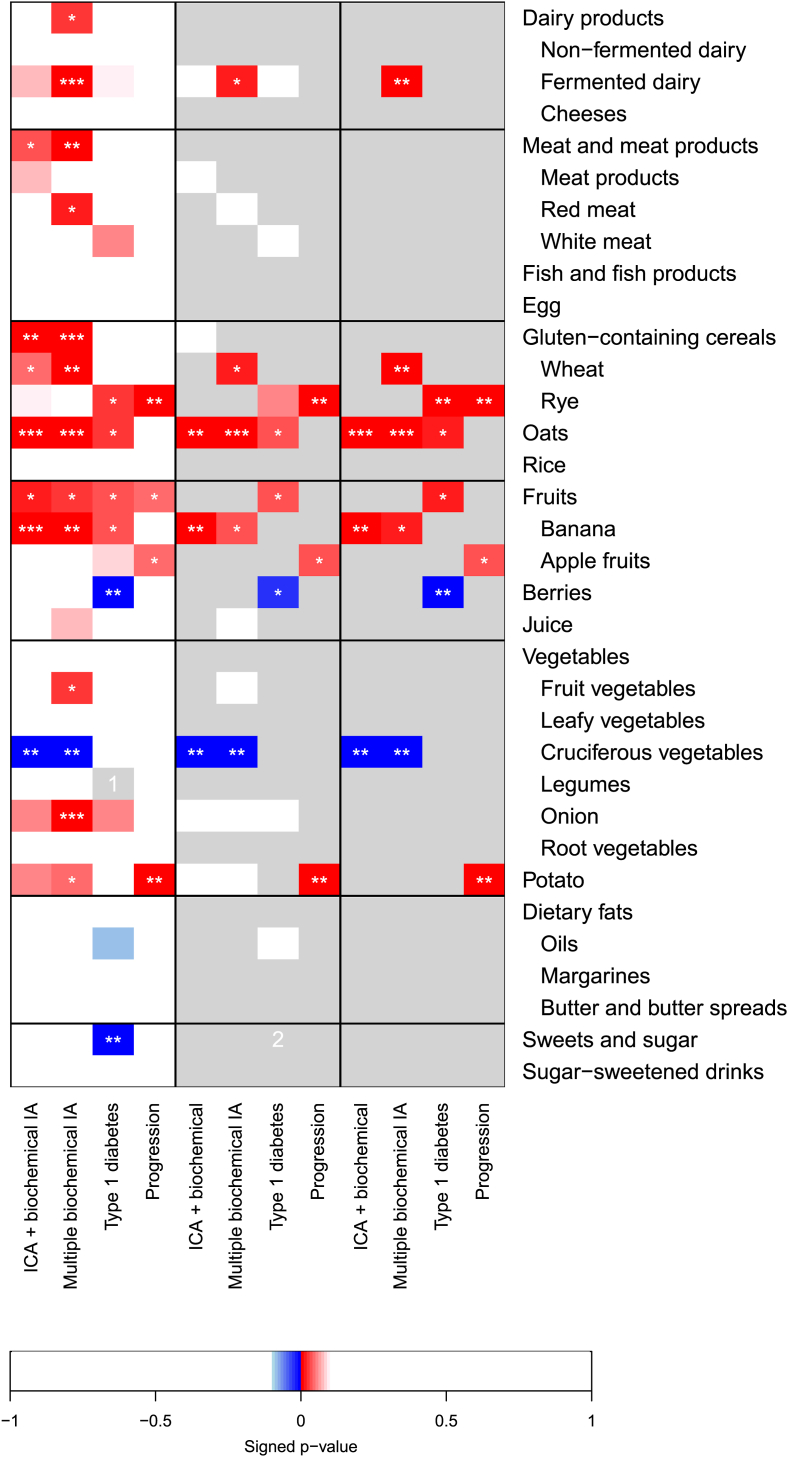
Summary of the associations between longitudinally assessed food consumption and risk of 4 type 1 diabetes-related endpoints based on joint modeling adjusted for child’s sex, HLA-DQB1-conferred risk, and familial diabetes as baseline variables and for energy. For energy adjustment and construction of the models, see the footnotes of TABLE 2, TABLE 3, TABLE 4 and Supplemental Table 4. Results are shown for each 1-food model (left panel), preliminary multi-food model (mid-panel), and final multi-food model (right panel) for each endpoint. Results are presented as signed *P* values where sign (direction) was set with respect to the direction of the corresponding estimate for the association with red color indicating positive (harmful) and blue color negative (protective) valued estimate, ∗*P* < 0.05, ∗∗*P* < 0.01, and ∗∗∗*P* < 0.001. White indicates *P*> 0.1. Gray indicates that the variable was not included in the model. IA, islet autoimmunity; ICA, islet cell antibodies; HLA-DQB1, human leukocyte antigen-DQ beta 1 isotype. ^1^One-food model for legumes did not converge. ^2^Preliminary models where sweets and sugar were included did not converge, and thus, the variable was excluded from the model and further analyses.

Of the 34 foods, the consumption of meat and meat products, gluten-containing cereals, wheat, rye, oats, fruits, banana, onions, and potato were direct, whereas cruciferous vegetables were inversely associated with ICA plus biochemical IA, and we chose them to further analysis (*P* < 0.1, Figure 1, Table 2). As both wheat and rye (the main gluten-containing cereals) showed associations with ICA plus biochemical IA, we used the gluten-containing cereals variable in further analysis. As for fruits, only banana was associated with the endpoint; we used only bananas in further analysis.

Once these foods were to the same model, oats and bananas remained directly and cruciferous vegetables inversely associated with the ICA plus biochemical IA endpoint (Figure 1, Table 2).

Adjustment for energy-adjusted protein or fat intake did not change the results. When energy-adjusted total dietary fiber was included in the model, the associations for both oats and bananas weakened while cruciferous vegetables remained significantly inversely associated with the endpoint (data not shown).

### Multiple biochemical IA

Of the 34 foods, 14 were selected for further analysis (Figure 1, Table 3). The consumption of dairy products, fermented dairy, meat and meat products, red meat, gluten-containing cereals, wheat, oats, fruits, bananas, juice, fruit vegetables, onions, and potatoes showed direct, whereas cruciferous vegetables showed an inverse association with multiple biochemical IA (*P* < 0.1, Figure 1, Table 3). As for dairy products, only fermented dairy, gluten-containing cereals, wheat and fruits, and only bananas were associated with the endpoint; we used only fermented dairy, wheat, and banana, respectively, in further analysis.

When adding all the before-described foods to the same final multi-food model, fermented dairy, wheat, oats, and banana remained directly, and cruciferous vegetables were inversely associated with the endpoint (Table 3).

Adjusting for energy-adjusted protein, fat, or n–3 fatty acid intake did not affect the associations. When energy-adjusted total dietary fiber was included in the model, the association for bananas weakened, whereas fermented dairy, wheat, oats, and cruciferous vegetables remained significantly associated with the endpoint (data not shown).

### Type 1 diabetes

Of the 34 foods, 10 were selected for further analysis (Table 4). The consumption of fermented dairy, white meat, rye, oats, fruits, bananas, apple fruits, and onions was directly, whereas berries, oils, and sweets were inversely associated with the risk of type 1 diabetes (*P* < 0.1, Figure 1, Table 4).

**TABLE 4:** Risk of type 1 diabetes by age 6 y (*n* = 94) associated with food consumption (*n* = 5674)^1^.

Energy and foods^2^: main groupsand subgroups	One-food models^2^		Preliminary multi-food model^3^		Final multi-food model^4^	
	HR (95% CI)	*P* value	HR (95% CI)	*P* value	HR (95% CI)	*P* value
Energy intake (MJ)	1.46 (1.10, 1.95)	0.008	1.47 (1.06, 2.01)	0.022	1.51 (1.11, 2.07)	0.010
Dairy products						
Fermented dairy (20 g/MJ)	1.53 (0.96, 2.19)	0.066	1.51 (0.90, 2.34)	0.111		
Meat and meat products						
White meat (1 g/MJ)	1.20 (1.02, 1.35)	0.030	1.12 (0.94, 1.31)	0.201		
Gluten-containing cereals						
Rye (1g/MJ)	1.22 (1.03, 1.43)	0.021	1.25 (1.04, 1.47)	0.022	1.27 (1.07, 1.50)	0.005
Oats (1 g/MJ)	1.12 (1.01, 1.21)	0.030	1.14 (1.03, 1.25)	0.019	1.15 (1.03, 1.26)	0.014
Fruits (1 g/MJ)	1.04 (1.00, 1.08)	0.036	1.05 (1.00, 1.08)	0.030	1.05 (1.01, 1.09)	0.011
Banana (1 g/MJ)	1.11 (1.01, 1.21)	0.031				
Apple fruits (1 g/MJ)	1.07 (0.99, 1.14)	0.081				
Berries (1 g/MJ)	0.60 (0.47, 0.89)	0.002	0.69 (0.52, 0.92)	0.003	0.67 (0.50, 0.93)	0.006
Vegetables						
Onions (1 g/MJ)	1.52 (1.00, 2.17)	0.051	1.32 (0.81, 2.04)	0.249		
Dietary fats						
Oils (250 mg/MJ)	0.83 (0.64, 1.02)	0.087	0.86 (0.66, 1.07)	0.201		
Sweets and sugar (1 g/MJ)	0.70 (0.52, 0.91)	0.008	5		5	

Abbreviations: CI, credible interval; HR, hazard ratio; MJ, megajoule.

1 HR and 95% CI are from the joint model with longitudinally assessed food consumption exposure (3 mo to 6 y). All the 1-food and multi-food models were adjusted for sex, genotype, and familial diabetes at baseline. In addition, the energy adjustment was done in all the models by dividing foods by energy and including energy in the model.

2 All the foods out of the 34 foods that were associated with the endpoint (*P* < 0.1) in 1-food models were selected for this table. Main food group names are presented for all the subgroups that remained in the analyses.

3 Foods significantly associated with the endpoint (P < 0.1) were entered into the preliminary multi-food model. If both the main food group and its subgroup were associated with the endpoint, the subgroup was used (did not occur in this table), but if several subgroups were associated with the endpoint, the main food group was used (e.g., fruits).

4 The final multi-food model included all the foods that were significant (*P* < 0.05) in the preliminary multi-food model and when included in the final model. Those variables that dropped from the analysis were further individually added to the model, but none of them was significantly (*P* < 0.05) associated with the endpoint (data not shown).

5 Sweets and sugar were excluded from the model because their model did not converge.

As both banana and apple fruits showed associations with the endpoint, we used fruits in further analysis. When including all the before-mentioned foods in the same model, rye, oats, and fruits remained directly, and berries were inversely associated with the endpoint (*P* < 0.05). The model, including sweets and sugar, did not converge.

Adjustment for energy-adjusted protein intake did not change the results, but after dietary fiber adjustment, only berries remained significant. After adjustment for energy-adjusted fat all the other associations remained except that the association with fruits became of borderline significance (data not shown).

In the sensitivity analysis excluding dietary measurements 18 mo before diagnosis of type 1 diabetes (14 children and 256 food records excluded), berries (HR: 0.09; 95% CI: 0.06, 0.13) and sweets (HR: 0.12; 95% CI: 0.05, 0.24) remained associated with the endpoint, whereas the association turned inverse for rye (HR: 0.07; 95% CI: 0.03, 0.13).

### Progression from IA to type 1 diabetes

The distribution of the participating children according to main baseline covariates and risk of progression from IA to type 1 diabetes associated with them are presented in Supplemental Table 3.

Among the 34 foods, 4 were selected for further analysis (Supplemental Table 4). Rye, fruits, apple fruits, and potatoes were directly (*P* < 0.1) associated with progression from IA to type 1 diabetes (Figure 1). As for fruits, only apple fruits were associated with the endpoint, so we took it to further analysis. Rye (HR: 1.51; 95% CI: 1.14, 2.00), apple fruits (HR: 1.12; 95% CI: 1.01, 1.24), and potatoes (HR: 1.09; 95% CI: 1.03, 1.16) remained associated with progression to type 1 diabetes in the final multi-food model (Figure 1, Supplemental Table 4). Adjustment for energy-adjusted fat or dietary fiber intake did not change the results (data not shown).

In the sensitivity analysis excluding dietary measurements 18 mo before diagnosis of type 1 diabetes (25 children and 240 food records excluded), apple fruits (HR: 1.23; 95% CI: 1.06, 1.44) and potatoes (HR: 1.13; 95% CI: 1.05, 1.23) remained significantly associated with the endpoint, whereas the association turned nonsignificant for rye (HR: 1.33; 95% CI: 0.65, 2.19).

### Additional adjustment and interaction analyses

Adjusting for breastfeeding (breastfed at the age of 6 mo), age at introduction of solid foods, or maternal education did not change the findings for foods regarding IA, type 1 diabetes, multiple biochemical IA, and progression endpoints. Interactions between a child’s sex and genetic risk with foods in the final models were observed only for the type 1 diabetes endpoint. The inverse association between berries and type 1 diabetes was stronger among boys (HR: 0.40; 95% CI: 0.21, 0.59) than girls (HR: 0.97; 95% CI: 0.75, 1.13, *P*-interaction < 0.001) and among those in the high (HR: 0.39; 95% CI: 0.21, 0.61) in comparison to those in the moderate genetic risk group (HR: 0.95; 95% CI: 0.62, 1.13, *P-*interaction < 0.001). No interactions between breastfeeding at 6 mo of age and consumption of foods were observed for any endpoint (data not shown). The criteria indicated some level of time-dependency in association with fermented milk products, wheat, oats, and cruciferous vegetables with multiple biochemical IA, and rye with type 1 diabetes (Supplemental Figure 2). However, the time-varying HR had mostly the same direction as the original HR, and its 95% CI included the original HR in all the models.

Figure 1 summarizes the associations of all 34 foods with the endpoints in the 1-food models (left panel), in preliminary multi-food models (mid panel) as well as in the final multi-food models (right panel). In the current study, cereal, fruit, and cruciferous vegetable consumption showed the most consistent and convincing (*P* < 0.01) associations with the development of type 1 diabetes. Introducing the fixed model consisting of energy-adjusted oats, gluten-containing cereals, fruits, and cruciferous vegetables to all the primary endpoints as a sensitivity analysis did not change the conclusions (Supplemental Table 5).

## Discussion

In multi-food models, higher consumption of cereals (oats or gluten-containing) and fruits were consistently associated with increased risk of all the IA and type 1 diabetes endpoints. On the contrary, higher consumption of cruciferous vegetables was associated with decreased risk of both the IA endpoints and that of berries with decreased risk of type 1 diabetes. In addition, in multi-food models, fermented dairy consumption was related to increased risk of multiple biochemical IA and that of potato with more frequent progression from IA to type 1 diabetes. Breastfeeding at 6 mo of age was associated with decreased risk of IA endpoints, but adjusting for that factor did not change the findings for foods regarding the IA and type 1 diabetes endpoints.

The DIPP birth cohort is a large, well-characterized study population with frequent exposure and endpoint measurements. The availability of the prospective longitudinal dietary data on the total diet of the child collected with an open method, which allows all possible foods and their combinations to be entered and processed, can be considered a major strength of this study. The openness of the food consumption method and standardized data collection and processing enabled us to consider changes over time in food habits and recipes. As important as the correct measurement of food consumption is the quality of the food composition database and its calculation algorithms. We could use a regularly updated national food composition database and were able to add recipes, for example, specific recipes of almost all commercial infant foods used were included. Special attention was paid to the correct classification of dairy [21], cereal [4], and vegetable and fruit [7,22] variables. The relatively high associations found in correlation and cross-classification analyses for the intakes of carotenoids, fatty acids, and their sources with the serum concentrations speak in favor of the high quality of the current dietary data [23,24].

The use of joint models for longitudinal and time-to-event data is another strength of this study. Compared with the traditionally used method, the Cox regression model, the joint model could map the relationship of the disease hazard to the underlying trajectory of the longitudinal food consumption. The consumption trajectories were linked to the time-to-event process of type 1 diabetes endpoints by the simultaneous fitting of 2 submodels, leading to unbiased and more efficient parameter estimates compared to the Cox model [25,26]. The use of a multivariate version of Bayesian joint models enabled a simultaneous investigation of multiple foods in relation to the endpoints. Previously, we have used a likelihood-based approach for the joint models, which did not allow for simultaneous analysis of multiple foods [[4], [5], [6]]. In the multivariate model, the result for each food was adjusted for other foods in the model in addition to selected confounding factors. As a result, we gained a deeper understanding of the role of childhood diet in the development of type 1 diabetes. It is noteworthy that many of the foods have not received research attention before. As far as we are aware, this is the first prospective study in humans to assess the associations between a large selection of foods (covering the entire diet) and IA or type 1 diabetes endpoints by a multivariate approach. An additional strength is that we were able to study the time-varying associations and detected only minor signals of time interactions.

Limitations of the study include that it cannot assess causality nor which components in studied foods would be associated with the IA or type 1 diabetes endpoints. Also, the children in the study carry genetic risk for type 1 diabetes, and it is unclear whether the findings can be generalized to the nonrisk population.

Our findings suggest that the previously reported [4,7] protective associations for berries and cruciferous vegetables and risk-increasing associations for cereals, fruits, and potatoes with the IA and type 1 diabetes endpoints are independent of each other and of other foods. Consumption of fermented dairy products was associated with multiple biochemical IA independently of other foods, whereas associations between dairy with other endpoints were weaker. These findings are partly in line with other prospective studies linking higher dairy consumption with a higher risk of IA/type 1 diabetes [3,6,8,12,27,28].

Foods include a variety of factors that could explain the observed associations. For oats and gluten-containing cereals, these factors could be cereal proteins, dietary fiber, α-amylase/trypsin inhibitors, advanced glycation end products, cereal mycobiota, toxins, heavy metals, or remnants of pesticides and fertilizers [4,29]. Examples for gluten of physiological mechanisms that could explain the proposed association are not known in humans, but animal models of type 1 diabetes suggest that gluten may affect gut permeability and gut microbiota and cause low-grade inflammation [29].

Compounds in vegetables and fruits that could increase the risk of type 1 diabetes development are pesticide residues, high starch and/or sugar content, or toxins such as those produced by streptomyces [7]. On the contrary, fruits, berries, and vegetables might have antiinflammatory, antioxidative, or gut microbiota-modulating properties mediated through, for example, vitamin C, polyphenols, or glucosinolates [7]. The observed protective association of cruciferous vegetables with IA is interesting as cruciferous vegetables-derived phytonutrients may have beneficial effects on the immune system and bacterial colonization through the aryl hydrocarbon receptor system [30]. It has been implicated that bovine insulin, milk fat, or milk protein could drive the associations between cow milk consumption and the development of type 1 diabetes [12,31]. Milk homogenization or heat treatment seems not to explain these associations [6].

Adjustment for dietary fiber weakened the associations of cereals and fruits with IA endpoints and type 1 diabetes suggesting that a common factor in fiber and in these foods may partly explain the findings. In the same way, dietary protein may partly explain the association between dairy products and multiple biochemical IA.

As several commonly consumed and nutritionally important foods relate independently of each other to the type 1 diabetes disease process in children, one should consider possible common denominators for these foods. Several environmental chemical exposures have been, although inconsistently, linked to the development of type 1 diabetes [32]. Of them, for example, some endocrine disrupting chemicals can be derived from foods. Certain chemicals may affect the immune system, and development of autoimmunity, and the function and survival of beta cells [32].

Also, diet may affect the gut microbiome, inflammation, permeability, and the gut’s ability to educate the immune system and consequently the individual susceptibility to immune-mediated diseases and further the development of or protection from dysbiosis and decreased microbial diversity [[33], [34], [35]].

These data should not be used for nutritional counseling. Many foods, for example, oats and rye, that are associated with increased risk of type 1 diabetes-related endpoints are considered as parts of a healthy diet.

In conclusion, in this study, the higher consumption of oats, gluten-containing cereals, and fruits was associated with increased cruciferous vegetables with decreased risk of several of the type 1 diabetes-related endpoints when considering all the foods in combination. Further etiological and mechanistic studies are warranted.

## Author contributions

The authors’ responsibilities were as follows – SMV, EJP, LH, SN, JI, JT, RV, MK, JN: concept and design; SMV, EJP, LH, SN, H-MT, SA, MÅ, JI, RV, MK, JN: acquisition, analysis, or interpretation of data; SMV, EJP, LH: drafting of the manuscript; EJP, H-MT, JN: statistical analysis; SMV, JT, RV, MK: obtained funding; SMV, EJP, LH, MÅ, SA, JI, JT, RV, MK, JN: administrative, technical, or material support; SMV, JI, RV, MK, JN: supervision; SA: data quality control; All authors performed critical revision of the manuscript for important intellectual content; All authors are responsible for the approval of the final version to be published; SMV, EJP: have full access to all data in the study and take responsibility for the integrity of the data and the accuracy of the data analysis; and all authors: read and approved the final manuscript.

## Funding

This study was supported by the Research Council of Finland (grants 63672, 68292, 79685, 79686, 80846, 114666, 126813, 129492, 139391, 201988, 210632, 250114, 276475, 308066, 339922); European Foundation for the Study of Diabetes award supported by EFSD/JDRF/Lilly; the Diabetes Research Foundation; the Juho Vainio Foundation; the Yrjö Jahnsson Foundation; Competitive Research Funding of the Turku and Oulu University Hospitals; the Competitive State Research Financing of the Expert Responsibility area of Tampere University Hospital (grants 9E082, 9F089, 9G087, 9H092, 9J147, 9K149, 9L042, 9L117, 9M036, 9M114, 9N086, 9P057, 9R055, 9S074, 9T072, 9U065, 9V072, 9X062, 9AA084, 9AB083); the Juvenile Diabetes Research Foundation (grants 4-1998-274, 4-1999-731, 4-2001-435, 1-SRA-2016-342-M-R, 1-SRA-2019-732-M-B, and 3-SRA-2020-955-S-B); the Novo Nordisk Foundation; the European Union Biomed 2 Program (BMH4-CT98-3314). The funding organizations had no role in study design, data collection, analysis, and interpretation, nor in writing and the decision to submit the report.

## Data availability

The data sets generated and analyzed during the current study are not publicly available due to the protection of the identity of the study participants and their clinical data. Data described in the manuscript, code book, and analytic code will be made available upon reasonable request (e.g., application and approval, payment, other).

## Conflict of interest

The authors report no conflicts of interest.
