# Supplementary material for: Food Consumption and Risk of Islet Autoimmunity and Type 1 Diabetes in Children at Increased Genetic Susceptibility for Type 1 Diabetes
Source: J Nutr. 2024 Sep 21;154(11):3465–74. doi: 10.1016/j.tjnut.2024.09.018 (PMC11600112; doi:10.1016/j.tjnut.2024.09.018)
Supplement: Multimedia component 1 [file mmc1.docx]

**Appendix A: Supplementary material**

Virtanen SM, et al. Food Consumption and Risk of Islet Autoimmunity and Type 1 Diabetes. The DIPP Birth Cohort.

**Supplementary Methods**

**Supplementary Tables**

**Supplementary Table 1** Foodss included in the endpoint analyses. These ingredient-based variables cover the entire diet of the participating children

**Supplementary Table 2** Risk of ICA plus biochemical islet autoimmunity (n = 247) and multiple biochemical islet autoimmunity (n = 206) by age 6 years associated with energy intake and absolute food consumption (n = 5626)

**Supplementary Table 3** Children with confirmed positivity to at least one islet autoantibody according to baseline characteristics and the risk of progression to type 1 diabetes by age 6 years

**Supplementary Table 4** Risk of progression from confirmed islet autoantibody positivity (n = 505) to type 1 diabetes by age 6 years (n = 64) associated with energy-adjusted food consumption

**Supplementary Table 5** Risk of islet autoimmunity and type 1 diabetes associated with multi-food model comprising gluten-containing cereals, oats, fruits, and cruciferous vegetables

**Supplementary Figures**

**Supplementary Figure 1** The flow chart of the DIPP study population

**Supplementary Figure 2** Visualization of the time-varying associations for the models with indication of time interaction

**Supplementary Methods**

**Study design and participants** For this study, 54 350 children born between September 1996 and September 2004 in Oulu and Tampere University Hospitals in Finland were screened from cord blood for genes indicating high or moderate HLA-conferred risk for type 1 diabetes as a part of the Type 1 Diabetes Prediction and Prevention (DIPP) prospective birth cohort study. Genotypes HLA-DQB1*02/*0302 represented “high” and HLA-DQB1 *0302/x “moderate" risk for type 1 diabetes. (x indicates alleles other than DQB1 *02, *0301, or *0602/3). Since April 1997 DQB1*0602/3 probe recognizing both DQB1*06:02 and DQB1*06:03 alleles was replaced by a probe specific for DQB1*06:02. Of the screened children, 8293 (15%) were eligible, 7782 were invited, and 6080 children (78% of those invited) participated in the islet autoimmunity (IA) follow-up (ESM Fig. 1). Children were screened for ICA at intervals of 3-12 months. After positivity for ICA for the first time, all preceding and subsequent samples from that participant were analyzed for biochemical autoantibodies: IAA, GADA, and IA-2A. ICA were quantified by a standard indirect immunofluorescence method, and IAA, GADA, and IA-2A with specific radio-binding assays.

Of the 6080 participants enrolled in the IA follow-up, 5626 children (93%) had food record and IA data available and of the 7782 children with type 1 diagnosis data 5674 children (73%) had food record data. The dropout rates among the 5674 participants at 1-, 2-, and 6-year follow-up were 8%, 16%, and 32%, respectively. The total numbers of food record days from 3 months to 6 years were 79954, 80126, and 81075 in the analyses with ICA plus biochemical IA, multiple biochemical IA, and type 1 diabetes endpoint, respectively. The diagnosis of type 1 diabetes by the age of 6 years was more common among the children from whom food record data was available compared to other children (1.7% vs. 0.9%, *P*=.008).

Of the participating children, 505 were detected repeatedly positive for at least one autoantibody and had also at least one food record available at or after seroconversion and before the diagnosis of type 1 diabetes and were included in the progression from IA to type 1 diabetes analysis.

**Dietary methods** The research nurses advised the families before recording how to complete food records during pre-specified mainly consecutive days (2 week and 1 weekend day) and checked the food records during the visits. The type of daycare, source of food, contact information, menus, and recipes were asked in detail from the daycare personnel. The families were asked to check the food records completed at daycare with the daycare personnel. Families and daycare personnel received written advice including an example of one day food record. In the advice to families, emphasis was put on the frequency and completeness of meals and snacks, on the amounts of foods and drinks, on food preparation methods, on the type and amount of dietary fats used in food preparation, in salads, and on bread, on the type of milks and milk products used, and on the type of breads used. To minimize reporting errors due to the recording process, we gave the recording dates for the families and daycare in advance and stressed that keeping the record should not influence what the child eats or drinks. Probing was used to get information on between-meal snacks, juices, sweets, dietary supplements, and other easily forgettable foods. Brand names were asked for all commercial infant foods, infant formulas, and dietary supplements. Continuously updated food product brochures and list of dietary supplements available helped in identification of foods and dietary supplements at the study centers. A food portion size booklet, household measures, and food product brochures were used for estimation of amounts of foods used. The research personnel who advised the families and checked the food records in the study centers as well as the nutritionists who entered the dietary data and called the families, daycare, and study centers received continuous training and motivation.

**Statistical methods** In joint models, the locations of knots were determined through an algorithm by fitting linear mixed effects (LME) models with all relevant combinations of knots and selecting the best fitting one based on the Bayesian information criterion. The baseline hazard was set as a piecewise constant with cut points at the ages of 1.99 and 3.99 years given the follow-up of 6 years. The standard default priors were used for the parameters. The Gelman-Rubin diagnostic (1) was used to assess the convergence of the Markov chain Monte Carlo sampler, with diagnostic values <1.1 considered as convergence. In the multivariate joint models, correlation between multiple foods was captured by assuming a multivariate normal distribution for the random effects.

In the secondary analyses with progression used as endpoint, the time of seroconversion was used as time origin. The food intakes measured at or after the origin were included in the LME model. Ages, and thus the intakes, at origin varied a lot (3 months to 6 years) between individuals, and no one had intake measured exactly at that time. To ensure the appropriate fit for the individual trajectories prior to first available measurement after origin, linear interpolation between measurement prior to (if available) and following the origin was used to impute the intake at origin. The baseline hazard was set as a piecewise constant with cut points at the times of 1.99 and 3.99 years given the follow-up from seroconversion until 6 years of age. Otherwise, the models for progression endpoint were similar to those assessing type 1 diabetes in the entire cohort.

References

1. Gelman A, Rubin DB. Inference from Iterative Simulation Using Multiple Sequences. Stat Sci. 1992;7(4):457–72.

**Supplementary Tables**

**Supplementary Table 1.** Foods included in the endpoint analyses. These ingredient-based variables cover the entire diet of the participating children

| **Foods that were analyzed for type 1 diabetes endpoints: main group, subgroups indented** | **The main foods included in food group** | **Average proportion of users, %** | | **Mean (SD) amount of intake of all, g** | |
| --- | --- | --- | --- | --- | --- |
|  |  | **3, 6, and 12 mo. (n = 5588)** | **2 to 6 years**  **(n = 4173)** | **3, 6, and 12 mo. (n = 5588)** | **2 to 6 years (n = 4173)** |
| Dairy products | non-fermented and fermented dairy products, cheeses | 68.7 | 97.7 | 381 (362) | 577 (288) |
| Non-fermented dairy | milk, cream, ice cream, milk powder, cow’s milk based infant formula | 68.3 | 96.9 | 361 (351) | 490 (277) |
| Fermented dairy | soured milk, fermented milk products, fermented cream products | 17.4 | 48.9 | 17.8 (51.7) | 72.5 (100) |
| Cheeses | ripened and un-ripened cheese | 11.2 | 57.7 | 1.6 (7.0) | 14.5 (21.9) |
| Meat and meat products | unprocessed and processed meat | 50.3 | 95.3 | 17.3 (25.8) | 72.9 (53.3) |
| Meat products | meat products, sausage | 4.0 | 55.9 | 1.0 (6.4) | 23.9 (37.9) |
| Red meat | beef, lamb, pork, offal, sausage | 40.3 | 84.7 | 12.8 (22.0) | 54.0 (49.1) |
| White meat | poultry | 15.6 | 25.7 | 3.9 (12.2) | 12.7 (30.8) |
| Fish and fish products | unprocessed and processed fish | 7.4 | 21.6 | 1 .7 (8.0) | 8.7 (22.3) |
| Egg |  | 9.7 | 59.9 | 0.8 (4.8) | 8.8 (17.1) |
| Gluten-containing cereals | wheat, rye, barley | 43.6 | 97.9 | 9.2 (16.7) | 58.8 (35.5) |
| Wheat |  | 42.1 | 97.5 | 5.8 (11.6) | 44.4 (31.6) |
| Rye |  | 26.2 | 73.2 | 2.4 (6.6) | 12.8 (15.0) |
| Oats |  | 47.5 | 59.8 | 8.0 (13.6) | 10.2 (15.4) |
| Rice |  | 27.2 | 44.1 | 3.5 (9.1) | 9.7 (17.5) |
| Fruits | banana, apple fruits, citrus fruits, grape, pineapple etc. | 55.1 | 68.3 | 29.1 (42.7) | 57.8 (74.6) |
| Banana |  | 18.3 | 26.6 | 8.0 (23.8) | 18.1 (39.7) |
| Apple fruits | apple, pear, plum, apricot, peach | 43.9 | 36.2 | 14.4 (25.3) | 19.4 (42.4) |
| Berries | strawberry, bilberry, raspberry, currants etc. | 36.3 | 53.2 | 8.7 (17.3) | 17.7 (34.6) |
| Juice | 100% fruit and vegetable juices | 12.3 | 32.4 | 3.5 (18.6) | 43.2 (98.0) |
| Vegetables | root vegetables, leafy vegetables, cabbages, onion, fruit vegetables, edible fungi | 68.8 | 98.1 | 65.6 (71.6) | 128.5 (88.2) |
| Fruit vegetables | tomato, cucumber etc. | 19.3 | 72.0 | 4.2 (14.2) | 28.6 (40.0) |
| Leafy vegetables | lettuce, spinach etc. | 19.6 | 30.8 | 2.9 (11.7) | 3.2 (9.7) |
| Cruciferous vegetables | white cabbage, broccoli, cauliflower etc. | 15.1 | 16.3 | 3.4 (11.6) | 2.9 (12.1) |
| Legumes | beans, peas etc. | 10.2 | 30.5 | 1.0 (4.3) | 3.1 (10.5) |
| Onion | onions, leeks etc. | 24.6 | 68.2 | 1.7 (4.6) | 6.8 (8.9) |
| Root vegetables | carrot, rutabaga, beetroot etc. | 52.4 | 51.8 | 16.1 (24.1) | 15.4 (25.6) |
| Potato |  | 64.9 | 83.6 | 37.2 (47.6) | 71.2 (66.8) |
| Dietary fats | oils, margarines, butter, butter spreads etc. | 58.5 | 99.0 | 3.6 (5.3) | 18.5 (12.5) |
| Oils |  | 55.3 | 81.7 | 2.5 (3.9) | 3.6 (4.5) |
| Margarines |  | 7.9 | 64.9 | 0.4 (2.1) | 6.7 (8.7) |
| Butter and butter spreads |  | 13.5 | 69.7 | 0.4 (1.8) | 4.7 (7.4) |
| Sweets and sugar | chocolate, confectionery, sugar and syrups, sugar products | 60.4 | 94.7 | 5.8 (7.7) | 23.0 (27.1) |
| Sugar-sweetened drinks |  | 6.0 | 73.3 | 6.4 (38.7) | 211 (213) |

**Supplementary Table 2.** Risk of ICA plus biochemical islet autoimmunity (n = 247) and multiple biochemical islet autoimmunity (n = 206) by age 6 years associated with energy intake and absolute food consumption (n = 5626)^1^

| **Energy and foods^2^: main groups and subgroups** | **ICA plus biochemical IA**^1^ | | **Multiple biochemical IA**^1^ | |
| --- | --- | --- | --- | --- |
|  | **HR (95% CI)** | ***P* Value** | **HR (95% CI)** | ***P* Value** |
| Energy intake (MJ) | 1.05 (0.87, 1.25) | .632 | 1.14 (0.93, 1.40) | .191 |
| Dairy products (100 g) | 1.06 (1.00, 1.12) | .067 | 1.11 (1.04, 1.18) | .003 |
| Non-fermented dairy (100 g) |  |  | 1.06 (0.99, 1.14) | .083 |
| Fermented dairy (100 g) |  |  | 1.61 (1.29, 1.99) | <.001 |
| Meat and meat products (10 g) |  |  | 1.07 (1.01, 1.14) | .016 |
| Red meat (10 g) |  |  | 1.08 (0.99, 1.16) | .072 |
| Gluten-containing cereals (10 g) |  |  | 1.10 (1.01, 1.19) | .028 |
| Wheat (10 g) |  |  | 1.11 (0.99, 1.25) | .074 |
| Rye (10 g) |  |  | 1.19 (0.97, 1.46) | .100 |
| Oats (10 g) | 1.19 (1.07, 1.31) | .005 | 1.24 (1.12, 1.37) | <.001 |
| Fruits (10 g) |  |  | 1.07 (1.02, 1.11) | .004 |
| Banana (10 g) | 1.14 (1.05, 1.23) | .005 | 1.17 (1.07, 1.28) | <.001 |
| Vegetables (10 g) |  |  | 1.07 (1.01, 1.12) | .022 |
| Cruciferous vegetables (10 g) | 0.63 (0.31, 0.97) | .036 | 0.36 (0.14, 0.81) | .006 |
| Onions (10 g) |  |  | 1.70 (1.13, 2.62) | .008 |
| Potato (10 g) | 1.06 (1.01, 1.11) | .009 | 1.08 (1.02, 1.13) | .005 |

Abbreviations: CI, credible interval; HR, hazard ratio; IA, islet autoimmunity; ICA, islet cell antibodies; MJ, megajoule.

^1^Energy intake and of the foods those with *P* <.1 presented. HR and 95% credible interval are from joint model with longitudinally assessed food consumption exposure (3 months to 6 years).

^2^Each food was analyzed separately and adjusted for sex, genotype, and familial diabetes at baseline.

**Supplementary Table 3.** Children with confirmed positivity to at least one islet autoantibody according to baseline characteristics and the risk of progression to type 1 diabetes by age 6 years

|  | **Cohort of seroconverted children (n=505)**^1^ | | |
| --- | --- | --- | --- |
| **Characteristic** | **All, No. (%)** | **Type 1 diabetes, No. (%)** | **HR (95% CI)**^2^ |
| Child sex, No. (%) |  |  |  |
| Boys | 285 (56.4) | 42 (14.7) | 1 |
| Girls | 220 (43.6) | 22 (10.0) | 0.66 (0.39, 1.11) |
| HLA-DQB1-conferred risk, No. (%) |  |  |  |
| Moderate | 389 (77.0) | 41 (10.5) | 1 |
| High | 116 (23.0) | 23 (19.8) | 2.19 (1.31, 3.67) |
| Familial diabetes, No. (%) |  |  |  |
| No | 452 (89.5) | 54 (11.9) | 1 |
| Yes | 43 (8.5) | 9 (20.9) | 1.80 (0.89, 3.65) |
| Missing information | 10 (2.0) | 1 (10.0) | 0.86 (0.12, 6.30) |
|  | **Median (Q_1_, Q_3_)** | |  |
| Age at seroconversion, years | 1.91 (1.24, 3.51) | 1.24 (0.76, 1.76) | 0.67 (0.49, 0.93) |

Abbreviation: CI, credible interval; HR, hazard ratio; HLA, human leukocyte antigen.

^1^The median (first and third quartile [Q1, Q3]) follow-up time for the 505 children with confirmed autoantibody positivity was 3.6 (2.5, 4.7) years. Out of them 64 children progressed to type 1 diabetes at the median (Q1, Q3) time of 2.6 (1.8, 3.5) years and at the median (Q1, Q3) age of 4.2 (3.0, 4.8) years.

^2^Estimates are from the Cox proportional hazards model including child sex, HLA-DQB1-conferred risk, familial diabetes, and age at seroconversion as baseline covariates.

**Supplementary Table 4.** Risk of progression from confirmed islet autoantibody positivity (n = 505) to type 1 diabetes by age 6 years (n = 64) associated with energy-adjusted food consumption^1^

| **Energy and foods^2^: main groups and subgroups** | **One-food models**^2^ | | **Final multi-food model**^3^ | |
| --- | --- | --- | --- | --- |
|  |  | |  | |
|  | **HR (95% CI)** | ***P* Value** | **HR (95% CI)** | ***P* Value** |
| Energy intake (MJ) | 1.35 (0.96, 1.86) | .087 | 1.50 (1.03, 2.18) | .035 |
| Gluten-containing cereals |  |  |  |  |
| Rye (1 g/MJ) | 1.47 (1.13, 1.87) | .008 | 1.51(1.14, 2.00) | .004 |
| Fruits (1g/MJ) | 1.05 (1.00, 1.09) | .049 |  |  |
| Apple fruit (1 g/MJ) | 1.11 (1.00, 1.23) | .043 | 1.12 (1.01, 1.24) | .037 |
| Potato (1 g/MJ) | 1.08 (1.03, 1.14) | .004 | 1.09 (1.03, 1.16) | .006 |

Abbreviations: CI, credible interval; HR, hazard ratio; MJ, megajoule.

^1^HR and 95% credible interval are from joint model with longitudinally assessed food consumption exposure (3 months to 6 years). The one-food and multi-food models were adjusted for sex, genotype, and familial diabetes at baseline. In addition, the energy adjustment was done in the models by dividing foods by energy and including energy in the model.

^2^ All the foods out of the 34 foods, which were associated with the endpoint (p<.1) in one-food models were selected for this table. Main food group names are presented for all the subgroups which remained in the analyses.

^3^Foods significantly associated with the endpoint (p<.1) were entered to the preliminary multi-food model. If both main food group and its subgroup were associated with the endpoint, subgroup was used (e.g., apple fruit), but if several subgroups were associated with the endpoint, main food group would have been used (did not occur in this table). As all the foods included in the preliminary model were significant (*P* <.05), the preliminary model was the same than the final multi-food model.

**Supplementary Table 5.** Risk of islet autoimmunity (IA) and type 1 diabetes associated with multi-food model comprising gluten-containing cereals, oats, fruits, and cruciferous vegetables^1^

| **Foods in multi-food model** | **ICA plus biochemical IA^2^** | ***P* value** | **Multiple biochemical IA^2^** | ***P* value** | **Type 1 diabetes^2^** | ***P* value** |
| --- | --- | --- | --- | --- | --- | --- |
|  | **HR (95% CI)** |  | **HR (95% CI)** |  | **HR (95% CI)** |  |
| Gluten-containing cereals (1 g/MJ) | 1.05 (1.00, 1.09) | .052 | 1.08 (1.03, 1.13) | .004 | 1.10 (1.00, 1.21) | .050 |
| Oats (1 g/MJ) | 1.09 (1.04, 1.14) | <.001 | 1.12 (1.06, 1.17) | <.001 | 1.13 (1.02, 1.24) | .021 |
| Fruits (1 g/MJ) | 1.02 (1.00, 1.04) | .041 | 1.02 (1.00, 1.04) | .071 | 1.05 (1.01, 1.08) | .027 |
| Cruciferous vegetables (1 g/MJ) | 0.82 (0.72, 0.94) | .002 | 0.81 (0.69, 0.94) | .005 | 0.88 (0.64, 1.10) | .428 |

Abbreviations: CI, credible interval; HR, hazard ratio; IA, islet autoimmunity; ICA, islet cell antibodies; MJ, megajoule.

^1^HR and 95% credible interval are from the joint models with longitudinally assessed food consumption exposure (3 months to 6 years).

^2^The multi-food model was adjusted for sex, genotype, age at seroconversion, and familial diabetes at baseline. In addition, the energy adjustment was done by dividing foods by energy and including energy in the model.

**Supplementary Figures**


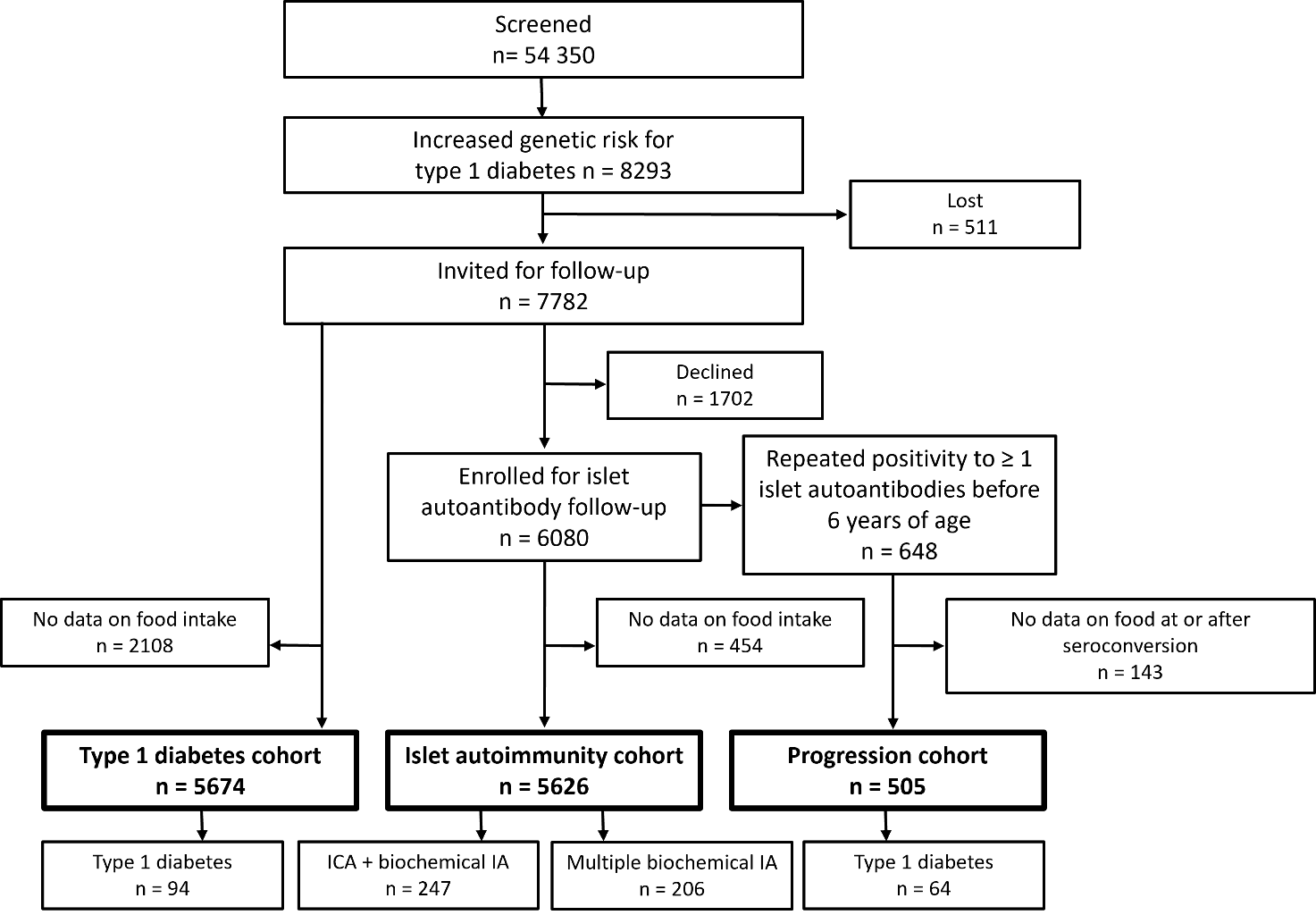


**Supplementary Figure 1:** The flow chart of the DIPP study population. IA, islet autoimmunity

*
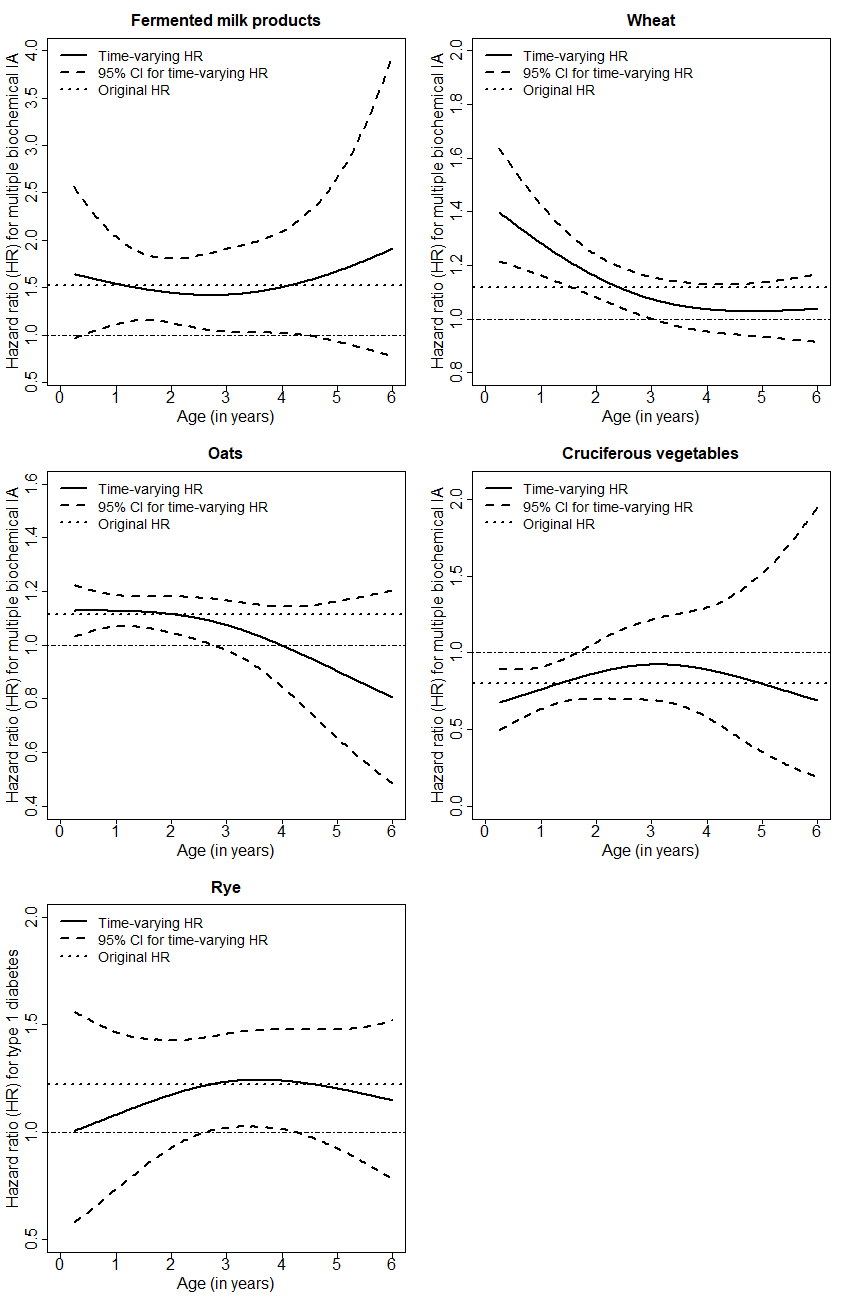
*

**Supplementary Figure 2:** Visualization of the time-varying associations for the models with indication of time interaction. Model for berries and type 1 diabetes did not converge. CI, credible interval; IA, islet autoimmunity
